# Ultra-resolution scalable microprinting

**DOI:** 10.1038/s41378-023-00537-9

**Published:** 2023-05-25

**Authors:** Callum Vidler, Kenneth Crozier, David Collins

**Affiliations:** 1 Department of Biomedical Engineering, University of Melbourne, Melbourne, VIC Australia; 2 School of Physics, University of Melbourne, Victoria, 3010 Australia; 3 Department of Electrical and Electronic Engineering, University of Melbourne, Victoria, 3010 Australia; 4 Australian Research Council (ARC) Centre of Excellence for Transformative Meta-Optical Systems, University of Melbourne, Victoria, 3010 Australia; 5 The Graeme Clark Institute, The University of Melbourne, Parkville, 3052 VIC Australia

**Keywords:** Engineering, Optical materials and structures

## Abstract

Projection micro stereolithography (PµSL) is a digital light processing (DLP) based printing technique for producing structured microparts. In this approach there is often a tradeoff between the largest object that can be printed and the minimum feature size, with higher resolution generally reducing the overall extent of the structure. The ability to produce structures with high spatial resolution and large overall volume, however, is immensely important for the creation of hierarchical materials, microfluidic devices and bioinspired constructs. In this work, we report a low-cost system with 1 µm optical resolution, representing the highest resolution system yet developed for the creation of micro-structured parts whose overall dimensions are nevertheless on the order of centimeters. To do so, we examine the limits at which PµSL can be applied at scale as a function of energy dosage, resin composition, cure depth and in-plane feature resolution. In doing so we develop a unique exposure composition approach that allows us to greatly improve the resolution of printed features. This ability to construct high-resolution scalable microstructures has the potential to accelerate advances in emerging areas, including 3D metamaterials, tissue engineering and bioinspired constructs.

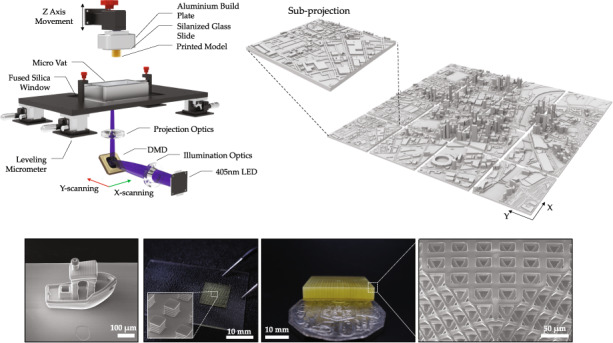

## Introduction

Recent advancements in additive manufacturing have enabled numerous application spaces to be explored when compared to traditional approaches. Such techniques include fused deposition modeling (FDM)^1,2^, direct ink writing (DIW)^3,4^, stereolithography (SLA)^5,6^, continuous liquid interface production (CLIP)^7–9^, computed axial lithography (CAL)^10–12^, xolography (XOLO)^13^ and two-photon polymerization (2PP)^14–16^, encompassing a range of physical principles and mechanisms. The majority of these excel at either macro-scale objects, with extents on the scale of cm’s, or micro-scales, with micron-scale resolutions and sub-mm scale print dimensions, where there is a dearth of approaches that can bridge the divide between these regimes. The need for such printing is evidenced by advances in meta-materials^17,18^, micro-optics^19,20^, biologically inspired constructs^21,22^, microfluidics^23^, acoustics^24^, and micro-electromechanical systems (MEMS)^25^ that could take advantage of the rapid, arbitrarily configured manufacture of microscale features at scale.

The techniques employed for the creation of micro and nano manufactured components depend on the feature size, material and overall dimensions of the desired micropart. A common method to produce microparts is two-photon polymerization, which relies on the non-linear absorption of the photoresist to contain polymerization to the focal volume^26,27^ The object is constructed by rapidly scanning this focal spot within a photosensitive material to sequentially cure the desired voxelized representation of the part, with sub-micron resolutions made possible by tuning the dimensions of this focal area^28^. Two-photon polymerization is often regarded as the gold standard for micro additive manufacturing, due to its excellent minimum achievable feature size and system flexibility. However, in practice the high system cost, mechanical complexity, high cost of consumables, and the small build volume limits the application space for 2PP.

Unlike 2PP, projection micro-stereolithography (PµSL) uses a digital micro-mirror device (DMD), liquid crystal display (LCD) or liquid crystal on silicon (LCoS) to produce patterned cross-sections of the desired part. These projections are then often pattered into a photopolymer either in a top-down approach^29^ or bottom-up^30^ to produce discrete two-dimensional cross-sections of the part which are therefore sequentially stacked to produce the desired three-dimensional object. Previous work has demonstrated applicability for projection micro-stereolithography for optical components^26^, tissue engineering^31^, microfluidics^32^, metamaterials^33^, 4D printing^34^ and biomedical applications^35^. Like 2PP, PµSL can be configured to produce voxel dimensions ranging from the order of optical wavelengths to tens of microns by controlling the numerical aperture of the imaging system and the degree to which the light is spatially confined in the *z* direction^36^. This can be accomplished by doping the resist or photopolymer with a material whose absorbance band similarly matches the initiation wavelength. In contrast to 2PP, PµSL offers some significant parallelization advantages as entire voxel grids can be written simultaneously. This approach has benefited from improvements in optical configuration^30^, resin development^37^, multi-material systems^38^ and slicing optimization^39^. However, PµSL is nevertheless subject to a tradeoff in terms of printing resolution and dimensions, as the size of the projected image gets smaller, the achievable volume must also shrink to accommodate it. Therefore, most high-resolution PµSL systems have relatively small build volumes, comparable to those found in 2PP, which greatly limits the types of objects constructed, their corresponding applications, and the number of parts that are simultaneously produced.

In this work, we have developed a compact projection micro-stereolithography system capable of producing micro-structures that span multiple length scales. Unlike previous techniques and implementations, our system enables ultra-high-resolution projection micro-stereolithography with an optical resolution of 1 µm, higher than any other such large-scale printing system to date, while nevertheless being able to generate structures that span tens of millimeters. In particular, this work explores the development and use of this Large Area Micro Printing (LAMP) system, including novel approaches for high-resolution slicing, exposure compensation and a custom resin system that together enable the generation of microscale features at scale. We further demonstrate this system in generating micron-scale structures, 3D objects with hollow microstructures, large pattered parts with complex topologies, and batch processing of microparts. The work demonstrates that scalable microprinting is an enabling tool for a wide range of discipline-spanning applications.

## System principles and characterization

**Fig. 1:**
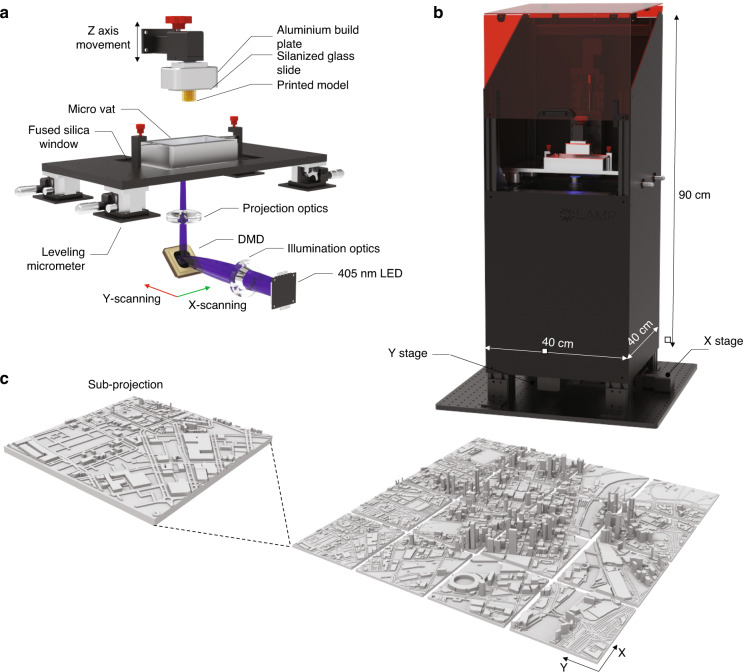
Large Area Micro-Printer (LAMP) system. **a** Schematic diagram of the optical and mechanical components of the ultra-resolution, 1 µm pixel size LAMP system. The XY scanning system traverses across the whole DLP and illumination assembly below the micro vat. **b** Model of the fully enclosed LAMP system. **c** Rendered model of the Melbourne central business district showing how the LAMP system segments large volumes into smaller sub-projections

Our PµSL system is configured in a bottom-mounted orientation (constrained surface technique). In contrast to a top mounted approach^40^, the bottom-up approach enables the precise control of the projection liquid interface, without the need for a wiping blade or free-surface control. In addition, the required resin volume is substantially lower and enables the use of a wider range of materials such as high viscosity polymers. A schematic representation of the components of the printing system can be found in Fig. 1. The Large Area Micro-Printer (LAMP) consists of a DLP light engine (Wintech, PRO6500) coupled to a 10X objective (NA = 0.28) to achieve an in-plane pixel size of 1 µm and a total single projection field of view of 1920 µm × 1080 µm. To accomplish fabrication of structures greater than the single projection window, the projection optics are mounted to a XY scanning stage (Optics Focus, MOXY-02-100-100-E) to facilitate in principle a maximum in-plane print surface of 100 mm × 100 mm. In addition, these stages facilitate the alignment of subsequent projected blocks with a precision of approximately 5 µm (Fig. S10). Structures are created within a micro-resin tank which consists of an aluminum CNC machined body with a transparent flexible window made from perfluoroalkoxy alkane (EPAX, X1 nFEP) film with a thickness of 100 µm. The resin tank sits on a floating fused silica window, which is positioned using four micrometer stages in each corner. This is used to level the bottom surface of the tank prior to printing using a high precision bubble level (Engineers Level, 61R-0.01-300). In addition, a custom motorized z-stage is connected to the projection optics to enable the precise measurement and control of the focal plane across the build volume. The build plate consists of a machined block which precisely holds a standard glass microscope slide, which is attached to a linear z stage (Optics Focus, MOX-02-100-E) located above the resin tank. A cross-section of the 3D object is created by scanning the projection optics and constraining the exposed layer between the build plate and the bottom of the build tank. By moving the *z*-axis up, this process is repeated for each desired object cross-section.

To limit ambient light and dust impacting the printing process, the LAMP is completely enclosed in a metal and acrylic chassis, with a sliding UV blocking lid to enable easy access. The LAMP system, which is schematically illustrated in Fig. 1, is designed to be relatively compact to enable it to fit on any standard lab bench space. DSLR images of the LAMP and its internal components can be found in Fig. S1. The approximate overall dimensions of the LAMP are 40 cm × 40 cm × 90 cm. In the following sections we detail the various approaches used to characterize and optimize the printing process.

### Vertical penetration characterization

**Fig. 2:**
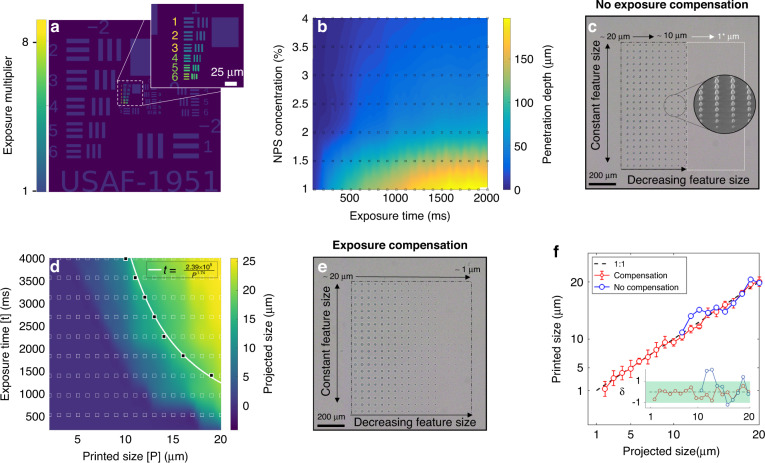
Development of exposure compensation approach. **a** Illustration of how the LAMP exposure compensation algorithm automatically segments regions of a given layer based on feature size. **b** Relationship between NPS concentration, exposure time and penetration depth. Black squares represent the measured data locations, the contour plot has been linearly interpolated between these locations to build a complete parameter space. **c** Array of circular features ranging from 20 → 1 μm (left to right) are projected without exposure compensation. Insert shows a HIM image of the test structure, with the white box indicating the region of unresolved features in the 10 → 1 μm range. **d** Exposure time represented as a function of feature size. White squares represent recorded data locations, black squares indicate the recorded approximate 1:1 mapping location. White line is the interpolated 1:1 mapping between the projected feature size and printed feature size based on the black squares. **e** Array of the same circular features from (**c**) with exposure compensation enabled. **f** Distribution plot of the produced in-plane feature size, with and without exposure compensation. Inset shows the maximum feature delta (*δ*) between the printed object and the projected feature measured in µm

A photo-absorbing compound is critical for creating accurate feature dimensions in the vertical direction (parallel to the optical axis), where the incorporation of 2-nitrophenyl phenyl sulfide (NPS) results in a concentration-dependent optical penetration depth. NPS was chosen due to its high solubility in PEGDA as well as having a high spectral absorption overlap with our 405 nm source. The concentration of NPS plays a key role for the creation of hollow or overhanging structures, such as those found in lattices and channels. Therefore, to investigate the role that this UV absorber plays in the minimum achievable layer thickness, a series of exposure tests were conducted for various exposure times and NPS concentrations. Figure 2b shows the parameter space explored for this study with the contour color corresponding to the penetration depth and the black squares representing the measured data locations. As expected, these results indicate that the penetration depth is dependent on NPS doping and scales approximately with the log of the exposure dose. For low concentrations of NPS (1%), the penetration depth ranges from 50–200 μm, comparable with many commercial based resins^41^. An example of the effect of NPS doping on vertical structural resolution, can be found in Fig. S2. However, at higher concentrations (4%), the penetration depth ranges 0.56–26.2 μm, enabling layer thicknesses to be controlled by modifying the exposure dose (from 100–2000 ms). The work reported by Gong et al., reported an NPS concentration of 2% to achieve similar penetration depths^42^. However, due to our inherently higher optical power density (enabling more rapid printing) a concentration of 4% was utilized for this work. Penetration depth was determined via optical profilometry of the exposure matrix to determine the minimum NPS doping needed to achieve sub-10-μm penetration depth. An example of optical profilometry measurements can be found in Fig. S3.

### Exposure compensation

Whereas the for small structures (<20 μm) the required exposure dose to resolve these features deviates significantly from that required for large structures, where the non-linear oxygen depletion threshold requires that the energy deposition scales (with the square) of the feature dimension. Therefore, the inclusion of this non-linear thresholding in the LAMP control software is imperative for pixel-scale features. We assessed this parameter space experimentally in building a dataset for an exposure compensation model, where a series of features ranging from (1–20 μm) were projected with increasing exposure time. An example of the segmentation method has been applied to the United States Air Force (USAF) test pattern as shown in Fig. 2a. These results developed a parameter space as seen in Fig. 2d, which represent the associated exposure dose needed to produce a given feature size. We note that a 1:1 mapping between the projected size and the printed size occurs along a line indicated in white. This mapping shows that the exposure time required to produce a feature <20 μm scales with approximately with α/P^2^, and the feature size for a given exposure dose increases approximately with $\beta \sqrt{1/t}$, where α and β represent resin-specific coefficients (listed in Fig. 2d). This model can then be used as the basis for the exposure compensation, whereby the size of a given feature determines the exposure utilized. The effect of enabling and disabling exposure compensation can be seen in a comparison between Fig. 2c and e, with the statical efficacy of exposure compensation being demonstrated in Fig. 2f. The exposure time without compensation was normalized such to produce an accurate 20 µm feature in-plane. Without compensation, features in the range of 20 µm → 10 µm showed significant deviation in size, with features <10 µm being unresolved. However, with exposure compensation enabled, feature recreation closely followed the idealized 1:1 mapping between object size and mask geometry, with an average error of ~1 µm. Using this approach features as small as 1.12 µm have been realized.

### In-plane feature characterization

**Fig. 3:**
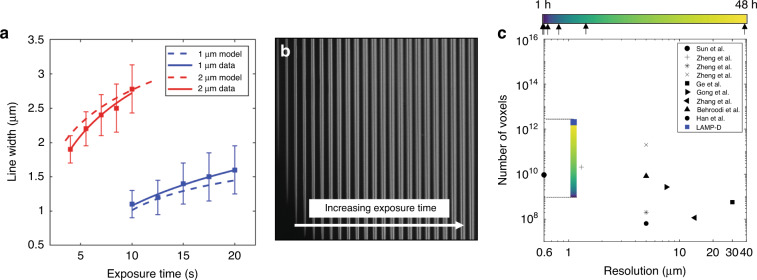
Exposure parameters and microscale feature dimensions, and comparison with prior work. **a** Line width measurements for 1 μm and 2 μm lines for increasing exposure time. **b** Helium ion microscopy (HIM) images of projected 2 μm lines imaged from above. **c** The LAMP printing system contrasted against prior projection micro-stereolithography based systems. Note that LAMP-D denotes the demonstrated maximum voxel number used in this work, evidencing the ability to produce large numbers of voxels at very high resolutions. Arrows below the color bar indicate the print times of select models. From left to right these include the mushrooms (Fig. 5c), benchy (Fig. 4a), CBML logo (Fig. 6b), microstructure array (Fig. 6g), Melbourne model (Fig. 8f) and periodic lattice (Fig. 8a)

A key feature of a microprinting system is the ability to resolve small features that are in proximity to each other. In many optical systems this characteristic is determined in part by the point spread function (PSF) and the modulation transfer function (MTF). This is further complicated by the local oxygen diffusion and kinetics of our resin system. Therefore, it is more relevant to assess these characteristics within the resist itself, rather than just of the optical system. This was investigated via a series of exposure tests conducted with 5 μm and 10 μm dots while implementing the exposure compensation model, highlighted in the previous section, to ensure that these features were correctly resolved. The edge to edge spacing between the dots was varied from 1 to 20 μm and were projected with varying exposure times. The results of this analysis can be seen Fig. S3, where we see that the exposure dose, feature spacing and individual feature size play a key role in the printer’s ability to differentiate features. In addition, helium ion microscopy (HIM) (Ziess, ORION NanoFab) images of the produced spots are shown in Fig. S4. Another key parameter is the printer’s capacity to create single pixel constructs, and to assess the features produced from single, 1 μm pixel exposures. As diffraction and scattering cause polymerization of neighboring domains, increased exposure time results in increased polymerized dimensions for a given exposure area. Further, oxygen diffusion results in a larger critical threshold for polymerization for smaller features than for larger ones. Fig. 3a exemplifies both of these characteristics, whereby optical exposures for 1 μm and 2 μm lines produce different printed dimensions for given exposures, and where smaller dimensions require a substantially greater initiation exposure dose to generate features. Once this critical energy dose is reached (*E*_*C*_), the line width scales with the log of the exposure energy, where our experimental results (solid lines) align with the line-width model (dashed lines) outlined in Eq. 3. An example of the produced lines can be seen in the HIM images in Fig. 3b.

## Results

### Importance of small pixel size

**Fig. 4:**
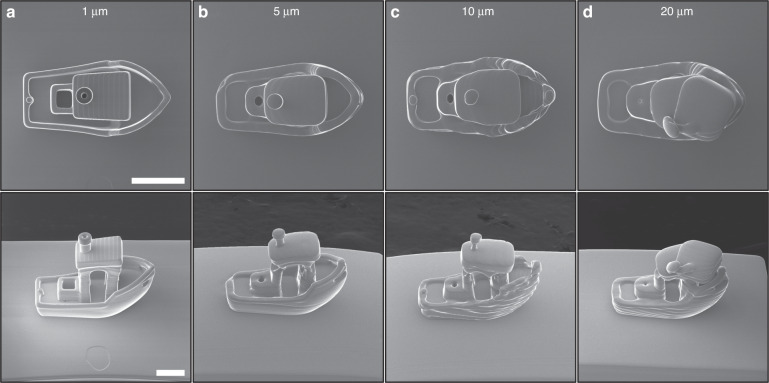
Importance of pixel size on achieved resolution illustrated using the 3DBenchy torture test. **a** 1 μm pixel size, **b** 5 μm pixel size, **c** 10 μm pixel size and **d** 20 μm pixel size. The projected masks for images (**b**–**d**) have been artificially down-sampled to produce the desired pixel size. Scale bars 100 μm

In contrast to the bulk of PµSL approaches^30,38^, our system produces voxel dimensions that are an order of magnitude smaller, while producing trillions of voxels within a printed construct. The predicted and experimental time taken to produce a given voxel number over a 48 h period can be found in (Fig. 3c). To highlight the impact that voxel resolution plays in producing high fidelity microparts, a 270 µm long, 140 µm wide 3DBenchy model (this model being widely used as a printing benchmark^43,44^ due to its complex 3D shape, overhangs, roofs, holes and curvilinear features) was printed with 1, 5, 10 and 20 µm pixel dimensions. To achieve effective pixel sizes larger than the native size defined by the optical setup (1 μm), the mask for each layer was artificially down-sampled to the desired pixel size for 5–20 µm pixels. The results of this comparison can be seen in Fig. 4 which highlight HIM images of the 3DBenchy for different in-plane pixel sizes, viewed from both the top (top row) and 45° from vertical (bottom row). In all these cases the layer height remained fixed at 2 μm with only the mask pixel dimensions being changed. Here an increasing pixel size results in the removal of high-resolution features, such as the hole in the chimney, but also an overall distorted shape and rougher surface topography due to the aliasing caused by the larger pixel sizes. This comparison highlights the unique advantage of utilizing smaller optical pixels to create detailed microparts that would be unprintable at the lower optical resolutions in other PµSL systems.

### Microstructure production

**Fig. 5:**
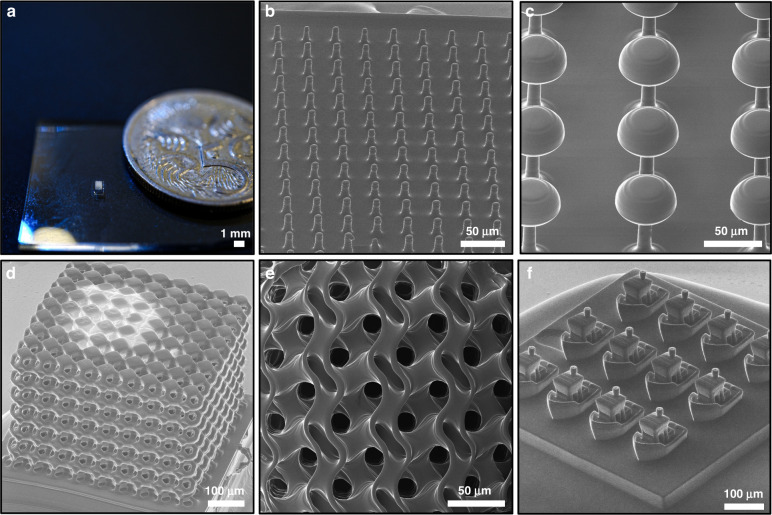
Single-projection (1 × 1 mm) parts. Parts imaged using DLSR (**a**) and HIM (**b**–**f**). **a** Kelvin-cell lattice imaged next to an Australian 5c coin, scale bar 1 mm. **b** Array of micropillars 10 μm in diameter and 40 μm tall. Scale bar 50 μm. **c** Bio-inspired mushroom structures, scale bar 50 μm. **d** Kelvin-cell lattice as shown in (**a**), scale bar 100 μm. **e** Top-down view of gyroid lattice, scale bar 50 μm. **d** 3DBenchy boat array, scale bar 100 μm

The LAMP system can be used in two primary modes depending on the size of the construct. In single-projection mode, the object size is dictated by the projector’s field of view. In multi-projection mode, the printer’s optics can be translated to create large objects via sequential stitching of single projections. Before extending to larger constructs, we first validate and characterize the ability for the printer to create objects that lie within a single projection window, as the overall achievable resolution is intrinsically dictated by the printer’s capacity to produce a single projection micro-part. We accordingly demonstrate the ability to produce a wide range of feature sets important for different application domains in Fig. 5, producing high-resolution micro-structures with complex topological makeups. Figure 5a highlights the scale of these single-projection constructs next to an Australian 5c coin, with each structure being 1 × 1 mm in area. Figure 5b–f demonstrates the ability to produce complex microstructures, with features as small as a few microns, including micropillars, mushroom structures with sharp overhangs, lattices and tiled 3DBenchy’s, where the boats in Fig. 5f are just ~100 µm wide. Further feature arrays and test prints can be found in Fig. S5 and Fig. S6.

**Fig. 6:**
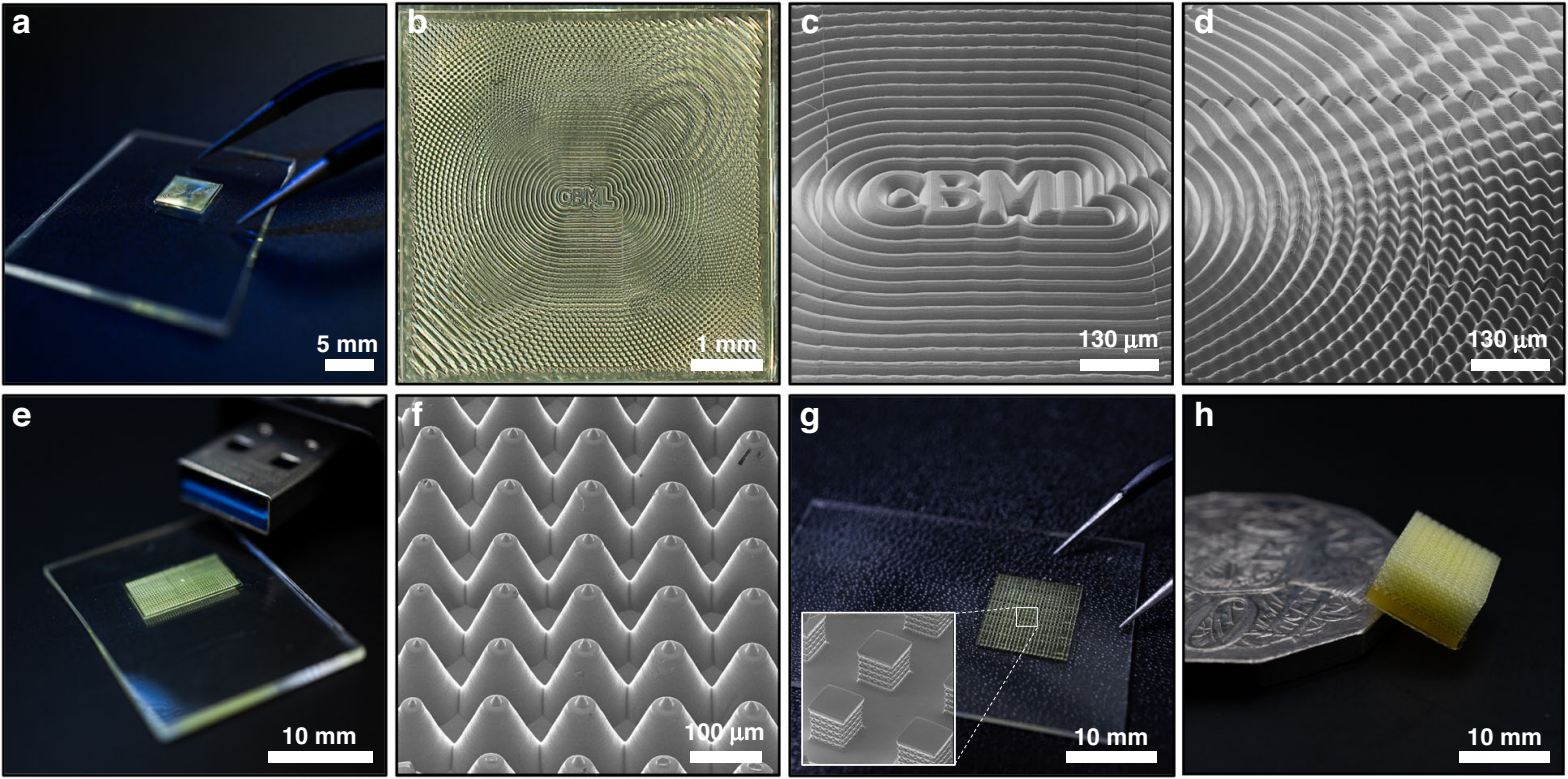
Parts produced using a multiple projection field of view. Parts imaged using DLSR (**a**, **e**, **g**), optical microscope (**b**) and HIM (**c**, **d**, **f**, **g**). **a** CBML lab logo print next to a pair of tweezers, scale bar 5 mm. **b** CBML lab logo print imaged under optical microscope, scale bar 1 mm. **c**, **d** Close-up image of surface topology and stitching error, scale bar 130 μm. **e** 10 × 5 mm microneedle array, scale bar 10 mm. **f** Close-up of microneedle array, scale bar 100 μm. **g** Array of 625 parts in a 10 × 10 mm area, scale bar 10 mm. **h** Micro-lattice next to an Australian 50c coin, scale bar 10 mm

This approach further incorporates the capacity to create high resolution features with 1 µm optical pixel dimensions across multiple projections. In the case of a single-projection field of view, the resolution of the micropart is determined by the resolution of the projected image and the layer height in the *z*-direction. However, for larger constructs the resolution is also dictated by the capacity to maintain image focus over large areas and accurately align stitch blocks onto neighboring regions. This is achieved by controlling the focus of the optical system as it moves between stitch locations, in addition to ensuring that there is sufficient mechanical overlap between neighboring stitch regions. Figure 6a–f illustrates a few large-scale stitched structures with length scales up to 10 mm. All of these structures still retain an in-plane optical resolution of 1 μm, and individual feature dimensions of a few microns. This is highlighted in the image of our lab logo Fig. 6c, d, in which the peaks of the ripples are approximately 4 μm. In addition, the secondary peak of at the tops of the micro-needle array shown in Fig. 6f incorporates features of 5 μm diameter.

Another key advantage of movable optics is the ability to batch process hundreds or potentially thousands of parts at a time. This is not only advantageous for reducing the cycle time in producing microparts, but can also enable the ability to batch process hundreds of different geometry configurations at once. The micropart array shown in Fig. 6g, contains 625 different variations of a gyroid lattice structure which were generated using nTopology (New York, NY, USA). This along with an automated nanoindentation setup, can, for instance, enable to quick and effective batch processing of micro lattice stiffness measurements.

**Fig. 7:**
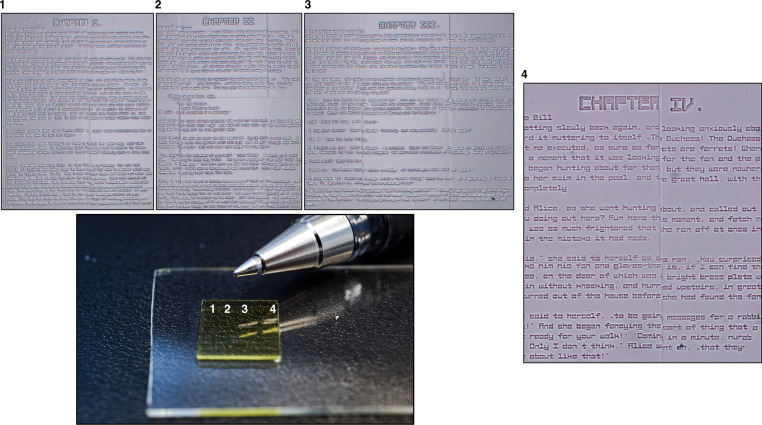
Alice in Wonderland. Numbered images correspond to different chapters of the Alice in Wonderland text and their associated placement on the 10 × 10 mm print. All text images were imaged using an inverted microscope, with the total text height for each letter being 40 μm

To further demonstrate the ability to produce large, non-periodic multi-stitch structures with a high degree of spatial resolution, the first 5 (of 12) chapters of Lewis Carroll’s Alice in Wonderland were printed onto the surface of a 10 × 10 mm domain, as shown in Fig. 7. The individual letters here are 40 μm tall, with 4 µm features (line widths), further demonstrating the production of high in-plane resolution across multiple stitched regions. The influence of stitching artifacts can be seen in images 1, 2 and 3, where a hatching overlap of 5 μm was used to ensure good mechanical adherence between neighboring blocks. The microtext structure is placed next to a pen tip for scale.

**Fig. 8:**
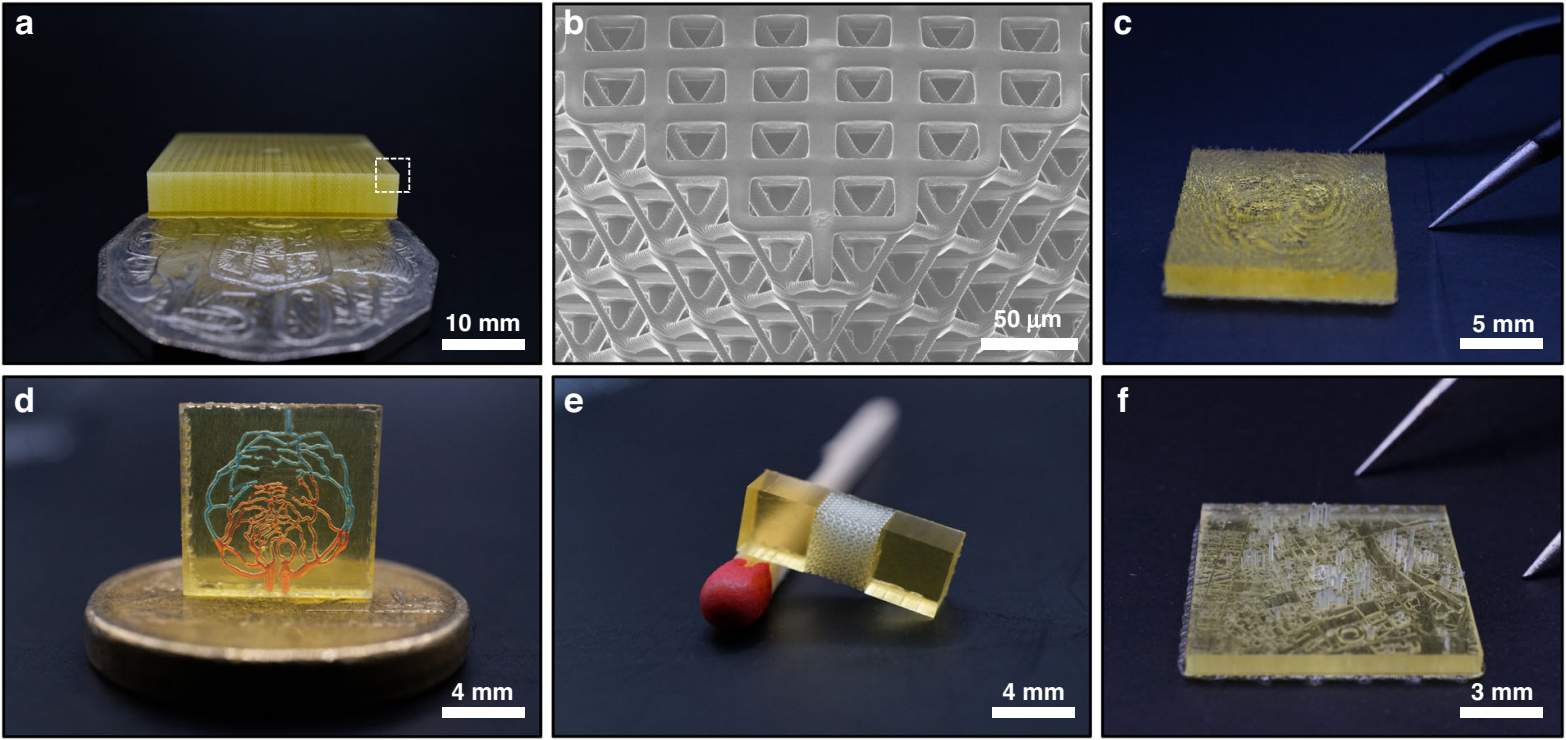
Examples of large, multi-stitched geometries. **a** 25 mm × 25 mm × 5 mm large periodic lattice with an Australian 50c coin for scale. **b** HIM of lattice shown in (**a**) in the region highlighted in white. **c** Non-periodic acoustic hologram, approximately 15 mm × 15 mm × 3 mm. **d** Neurovascular model with 100 μm channels containing red and blue dye. **e** Micro-lattice tensile sample shown against a matchstick. **f** Model of Melbourne central business district, containing all roads, buildings and bridges

Evidencing the ability to generate large-scale 3D prints, we further produce engineering-relevant topographies via a series of different prints, shown in Fig. 8. Large periodic lattices like those shown in Fig. 8a, b can be printed in less that 48 h and contain approximately 1.2 × 10^12^ voxels. A novel threshold which is immensely important for the creation of biologically relevant structures^45^. In addition, this approach can be used to produce non-periodic acoustic holograms^46,47^, as well as microfluidic devices, tensile test samples and high-aspect ratio structures Fig. 8c–f, with Fig. 8f for instance being a print of the city of Melbourne central business district.

## Conclusion

In this work, we develop a versatile microprinting platform for the printing of high resolution microparts across multiple projection domains, utilizing the highest yet optical resolution (1 µm) in such a system, and implementing a suite of exposure and resin optimizations to take advantage of this while highlighting the importance of this resolution for micropart printing. The resulting system is compact, enabling it to be placed on a standard lab bench, and cost-effective when compared to (lower resolution) commercial systems. In addition, we developed an exposure compensation approach capable of rendering very high-resolution cross-sections, without severe memory detriment via the use of scalable vector graphics (see Methods). In addition, a high-resolution resin formulation was optimized for ~0.5–20 µm layer thicknesses. An in-depth understanding of the parameter space that affects both the in-plane and out-of-plane resolution of the printing system was further explored, characterized and implemented into the slicing algorithm. We accordingly demonstrated a LAMP system capable of producing both high resolution single projection microparts, with the further ability to produce large complex structures that span multiple projections and dimensions on the order of centimeters. Moving forward, improvements in the scanning speed or projected field-of-view could enable the creation of teravoxel structures in less than 24 h, greatly enabling future applications in bioprinting, microfluidics and meta-materials. In addition, the use of a multi-objective system could facilitate the patterning of low-resolution structures containing high resolution features very quickly, by only using a high-resolution objective when needed. Furthermore, optical feedback to improve block alignment would greatly negate stitching artifacts located at block boundaries, further improving resolution and mechanical homogeneity. To further elaborate on the compatibility of the LAMP system with various resin-based constructs, it is worth noting that, in principle, any resin-based construct should be compatible with LAMP, provided that any contained particulates are below the size of the pixels and compatible with photoabsorbers. This feature significantly broadens the range of applications of the LAMP system, allowing for the fabrication of various microparts and structures with high resolution and scalability. Furthermore, the optimized high-resolution resin formulation used in the LAMP system can be broadened to accommodate different materials or applications including hydrogels^48^, ceramic-loaded composites^49^, and metal-loaded resins^50^. Overall, the compatibility of the LAMP system with various resin-based constructs and the flexibility in material selection make it a powerful tool for various research and engineering applications.

## Materials and methods

### Resin formulation

#### Chemicals

Phenylbis(2,4,6-trimethylbenzoyl)phosphine oxide (Igracure 819), 2-Nitrophenyl phenyl sulfide (NPS), Poly(ethylene glycol) diacrylate (PEDGA, Mn = 700), Isopropanol. All chemicals were purchased from Sigma Aldrich.

#### Resin formulation

The resin formulation used in this work consisted of Poly(ethylene glycol) diacrylate as the monomer, the photoinitiator (Igracure 819) and NPS as the UV absorber (which is used to precisely control the penetration depth). The resin was prepared by combining 0.2% w/w of Igracure 819 in PEGDA with varying amounts of NPS depending on the required penetration depth (1%, 2%, 3%, 4% w/w). To produce a homogeneous mixture the resin solution was heated to 50 °C and mechanically stirred for 30 min in a dark environment. After mixing, the solution was briefly degassed in a vacuum chamber to remove any trapped air bubbles. The resin was then stored in an amber airtight container for up to a few months. Printed parts were washed in isopropanol for 5 min and then either air-dried or dried under nitrogen

### Surface treatment

#### Chemicals

Toluene anhydrous 99.8, 3-(Trimethoxysilyl)propyl methacrylate (TMSPM), Isopropanol. All chemicals were purchased from Sigma Aldrich.

All 3D printed parts were printed directly onto a consumable glass slide for easy transport and storage without the risk of damaging the micropart. To ensure that the 3D printed part adheres well to the glass slide, the surface of the slide was silanized with TMSPM to enable a strong chemical bond between the polymerized resin and the glass surface. The glass slide was first sonicated in an Isopropanol bath for 5 min, followed by a 5 min sonication in distilled water. The slides were then dried using compressed air and submerged in a solution of Toluene-TMSPM solution (90/10 w/w) for 2 h. Finally, the treated slides were washed with distilled water and stored under Isopropanol for up to 2 weeks. Prior to printing the slides were dried under a stream of nitrogen and adhered to the build plate using a double-sided adhesive.

### Focusing method

Unlike previous techniques that use a camera system to determine the optimal focus at the image plane^29,30^, our system employs an iterative approach to determine the set of optimal z positions across the entirety of the vat. To accomplish this, a precision coverslip (Thorlabs, CG00K1) acting as a spacer was placed on top of the fused silica window and a droplet of resin was placed on the coverslip to mimic the PFA film spacing. The printer therefore moves to each corner of the desired build volume (e.g. 20 mm × 20 mm) and projects a series of patterns whilst moving the projection optics through a series of z moves. This consequently creates a set of images at the four corners of the build volume with each in-focus image corresponding to a different z-location. The coverslip is then imaged using an inverted microscope (Olympus, IXplore Standard) and the best focus from each four corners is selected. These offsets are hence entered into the control software to generate a contour map of the projection windows surface. This is used to adjust the relative z-position depending on the objective’s location across the vat. In addition, to ensure that the projected image is not affected by external vibrations during the scanning procedure an accelerometer was mounted to the side of the objective lens to continuously monitor the induced environmental and internal vibrations caused by the stage movement. Before each block is projected, the code checks to see if the vibration has settled below a certain threshold, thus minimizing vibration induced aberrations in the object.

### Feature characterization

The resolution of the printer is comprised of two primary components the in-plane resolution (*x-y*) which is in-part dictated by the projected resolution and the out-of-plane resolution (*z*) which is determined by the penetration depth of the resin.

#### Out of plane resolution

As reported by^51^ the maximum cure depth (*C*_*d*_) is a function of the natural logarithm of the exposure energy per unit area as defined in Eq. 1.

1 $${C}_{d}={D}_{p}{\rm{ln}}\left(\frac{{E}_{\max }}{{E}_{c}}\right)={D}_{p}{\rm{ln}}\left(\frac{t}{{T}_{c}}\right)$$

where *E*_*c*_ and *T*_*c*_ are the critical exposure energy and time required to start the gelation process and is denoted when *E*_max_ > *E*_*c*_. Furthermore, the penetration depth into the resin *D*_*p*_ is defined by Eq. 2.

2 $${D}_{p}=\frac{1}{{\epsilon }_{d}\left[D\right]+{\epsilon }_{i}[S]}$$

where *ϵ*_*d*_ and *ϵ*_*i*_ denote the molar absorption coefficients of the photo initiator and UV absorber respectively. *D* and *S* denote the concentrations of the photo initiator and UV absorber respectively. *D*_*p*_ is often also defined as the depth of resin in which will reduce the irradiance to 1/*e* of the surface irradiance. As the concentration of UV absorber is significantly higher than that of the photo initiator, the penetration depth (*D*_*P*_) is dominated primarily by NPS. As the cure depth (*C*_*d*_) is pivotal in creating fine microstructures, the concentration of NPS and exposure time was varied to determine the optimal parameter space. To achieve this a droplet of resin was placed onto a silanized coverslip and a series of 100 µm × 100 µm squares were exposed with varying exposure times ranging from 100 ms–2000 ms. This was repeated for different concentrations of NPS ranging from 1 to 4% (w/w). The slides were then washed to remove non-polymerized resin using an isopropyl bath and dried under nitrogen. The heights of the exposed squares were measured using an optical profilometer (Bruker, ContourX-500) and averaged over the 100 µm × 100 µm area. Note that regions between the measured data locations were interpolated to ensure a continuous map of the parameter space. The optical power density used in this work was 141.5 mW/cm^2^ as measured using an optical power meter (Thorlabs, PM100D). Note that the LAMP optical power can be varied from ~50–175 mW/cm^2^ within the control software. The power response curve can be found, and its associated mapping can be found in Fig. S7.

### In-plane resolution

The in-plane resolution of the printing system is dependent on its optical characteristics as well as the features of the resin system. The parabolic cylinder formed by a projected line exhibits a maximum string width at the resins surface defined by the following relationship Eq. 3.

3 $${L}_{w}=\sqrt{2}{w}_{0}{{\rm{ln}}\left(\frac{{E}_{\max }}{{E}_{c}}\right)}^{0.5}={w}_{0}\sqrt{{C}_{d}/2{D}_{p}}$$

where *w*_0_ denotes the radius of the spot. The above relationship is immensely important as it indicates that the in-plane resolution of a filament is not only dependent on the spot size, but the resins penetration depth. To characterize the minimum achievable linewidth and spot size a series of single pixel lines and spots were exposed onto a silanized slide using a similar methodology as above. The exposure time and NPS concentration was varied to build a map of the parameter space.

### Exposure compensation

Like with almost all acrylate-based resin systems, oxygen inhibition constraints the rate in which polymerization can occur, with localized oxygen around the feature needing to first be depleted. As the projected structure gets smaller, the delivered power scales with the square of the area. This results in a challenging problem where smaller features require a different exposure dose, compared to larger features. To alleviate this issue, an exposure compensation routine is built into the print software that automatically detects and isolates regions within the mask depending on their size. These separated regions are consequently given a different exposure dose depending on their size (1–20 µm) in increments of 1 µm. This process is done on-the-fly for each new mask image and therefore does not require the masks to be pre-processed prior to printing.

To accompany this optimization a series of exposure tests were conducted which aimed to understand the parameter space in which the optimization operates. Using the same methodology as outlined in the feature characterization section, a series of spots ranging from 1 to 20 µm were exposed with varying exposure times. The corresponding size-time relationship was used as the basis for the exposure compensation and is shown in Fig. 2.

### LAMP control software

The LAMP control software enables full control over the micro-printer and includes features such as slicing, print inspection, printer control and a graphical user interface (GUI) built within the MATLAB programming environment. The user loads in an STL which is converted to a series of 2D slices which are stored in a scalable vector graphics format (SVG). This enables the slices to be scaled arbitrarily, without requiring large amounts of memory. This is specifically crucial for large parts which span tens of millimeters or for structures with small layer thicknesses. If the size of the desired cannot be created using a single projection field-of-view, the software will automatically determine the number of ‘blocks’ required to create the part and the required minimum path for each layer. In addition, the control software enables the user to define key print parameters such as exposure time, layer height, enabling/disabling image compensation, movement velocity, stitch compensation, and so on. The process flow breakdown of the printing software can be found in Fig. S8. In addition, the software calculates the expected print duration depending on a number of key parameters defined in the control software. The effect of these parameters on the total print time can be found in Fig. S9.

### Imaging methods

To facilitate imaging of small structures without the need of a conductive coating all microparts were imaged using the Ziess Orion NanoFab. The NanoFab was operated using the helium source and the flood-gun was used to actively neutralize the surface during imaging. All structures were imaged using an accelerating voltage of 30 kV, beam current of between 1 and 2pA and a field of view between 1000 and 20 µm depending on the size of the structure and region of interest. Structures that were printed directly onto the silanized slide were mounted to the stage using the integrated mounting clips, whereas structures that were printed directly on the metal substrate were mounted directly to the stage using double-sided carbon tape. All off-axis images were either imaged at 45° or 55° depending on the structure and region of interest.

Inverted microscope images were performed using the Olympus IXplore Standard using either the 4× or 10× objective.

All DSLR images of the microparts were taken using a Sony Alpha 7 III with a Sony E 30 mm F3.5 Macro Lens (Sony, SEL30M35) and were taken within a lightbox to ensure adequate and uniform illumination of the objects.

## Supplementary information

Supplementary Movie 1

Supplementary Information

## Data Availability

All data needed to evaluate the conclusions in the paper are present in the paper and/or the Supplementary Materials.
