# Supplementary material for: Ultra-resolution scalable microprinting
Source: Microsyst Nanoeng. 2023 May 25;9:67. doi: 10.1038/s41378-023-00537-9 (PMC10212948; doi:10.1038/s41378-023-00537-9)
Supplement: Supplementary file 2 — Supplementary Information [file 41378_2023_537_MOESM2_ESM.docx]

**Ultra-resolution scalable microprinting**

**Callum Vidler^1^, Kenneth Crozier^2,3,4^, David Collins^1,5*^**

^1^Department of Biomedical Engineering, University of Melbourne, Melbourne, Victoria, Australia

^2^School of Physics, University of Melbourne, Victoria 3010, Australia

^3^Department of Electrical and Electronic Engineering, University of Melbourne, Victoria 3010, Australia

^4^Australian Research Council (ARC) Centre of Excellence for Transformative Meta-Optical Systems, University of Melbourne, Victoria 3010, Australia

^5^The Graeme Clark Institute, The University of Melbourne, Parkville 3052, Victoria, Australia

*****Corresponding author

E-mail: [david.collins@unimelb.edu.au](mailto:david.collins@unimelb.edu.au)

**This PDF file includes:**

Supplementary Text

Figs. S1 to S10

**Other Supplementary Materials for this manuscript include the following:**

Movies S1 to S1


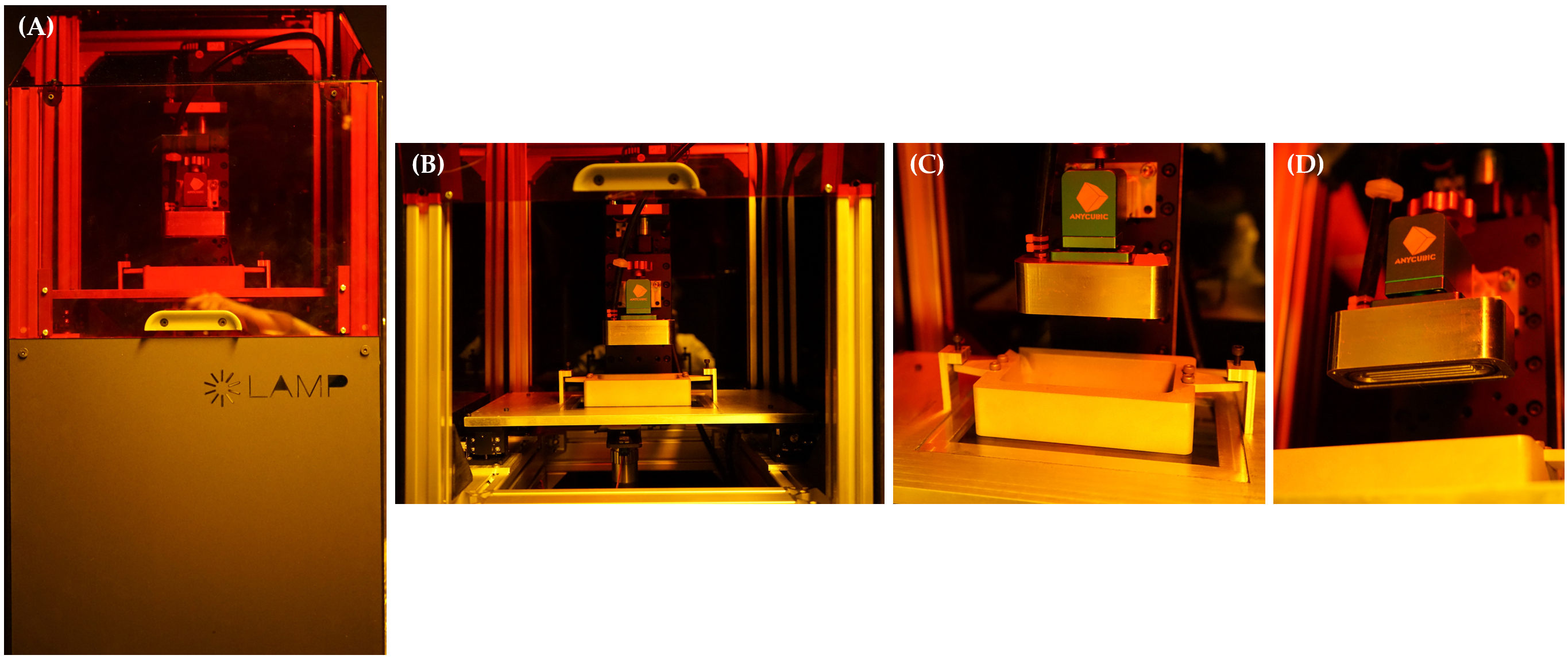


Fig. S1. DSLR images of LAMP printing system. (A) front view. (B-D) internal view showing the floating plate, micro vat and print bed assembly.


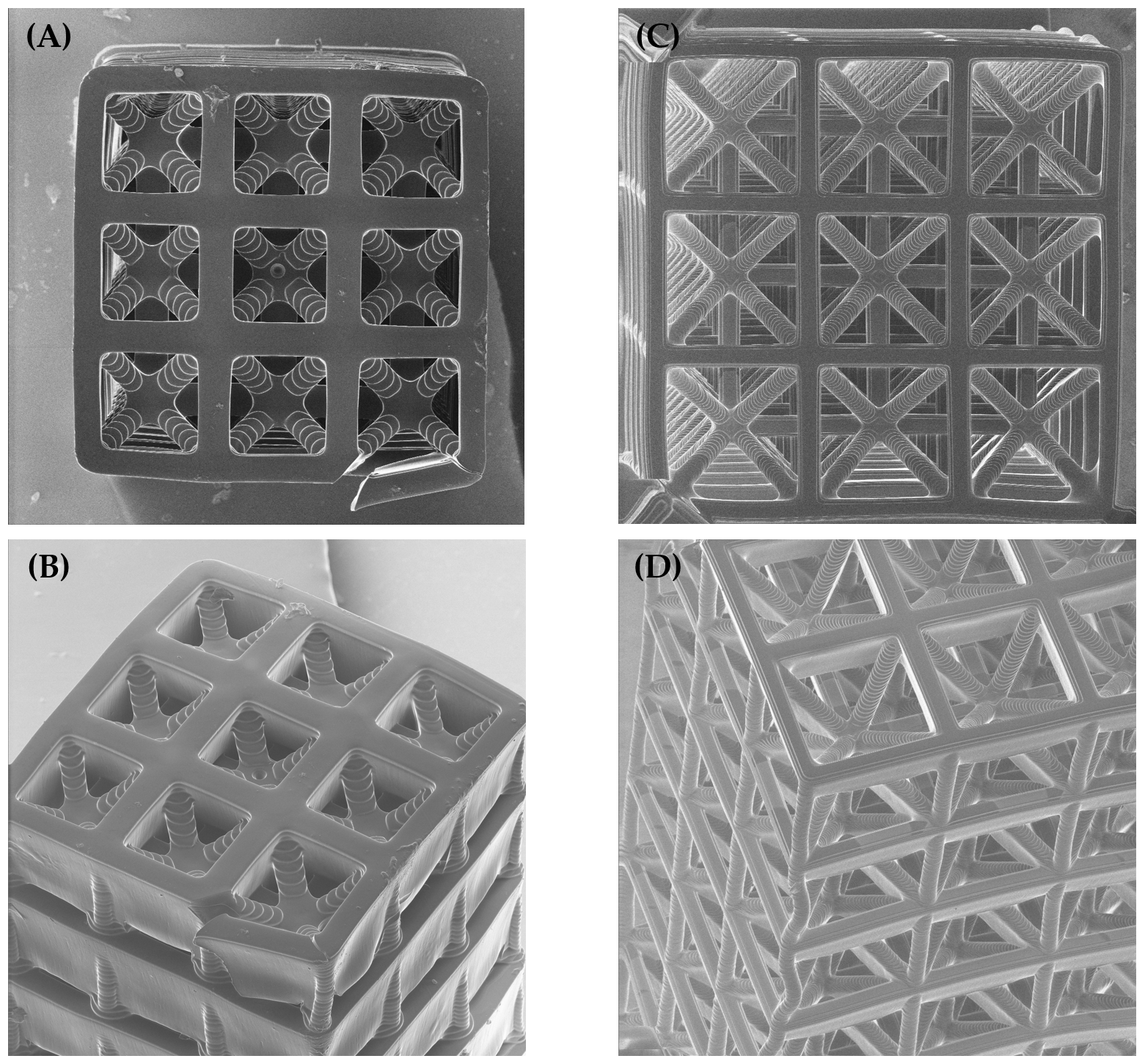


Fig. S2. Effect of NPS doping on vertical resolution. (A-B) PEGDA without NPS. (C-D) PEGDA formulation with 4% NPS. Each strut in the lattice is approximately 20 μm


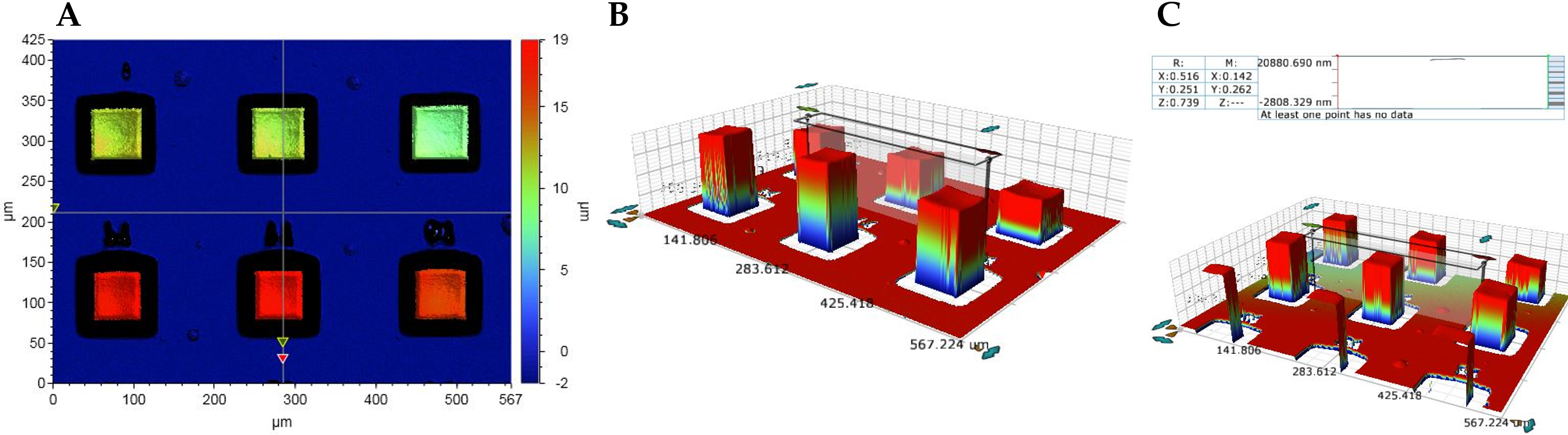


Fig. S3. Example of optical profilometry measurements for 4% NPS concentration. (A) Top view of the exposure dose array corresponding to penetration depths from ~ 6 – 19 μm. Each exposure pad is 100 µm x 100 µm, with the exposures in this region corresponding to approximately 460 ms – 980 ms. Colormap represents the height of the pillars in microns. (B) Side view of A. (C) Side view of the middle region of the exposure array.


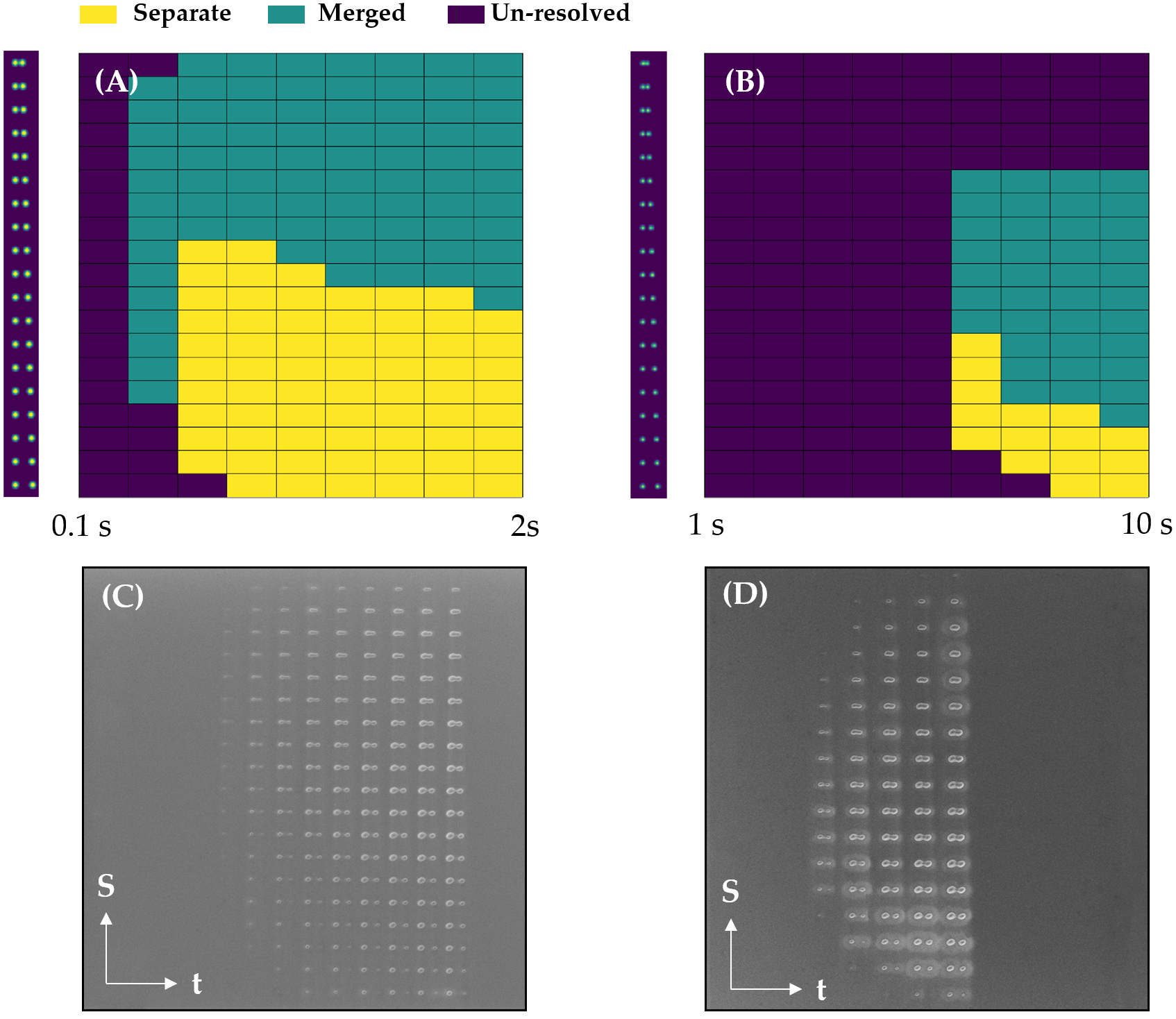


Fig. S4. In plane feature characteristics. (A) spot proximity measurements for 10 μm spots with a feature spacing ranging from 1-20 μm. The colours yellow, turquoise and purple represent separated, merged or no-data respectively. (B) spot proximity measurements for 5 μm spots. (C-D) represent helium ion images of the two configurations, with *S* and *t* representing decreasing spacing and increasing time respectively.


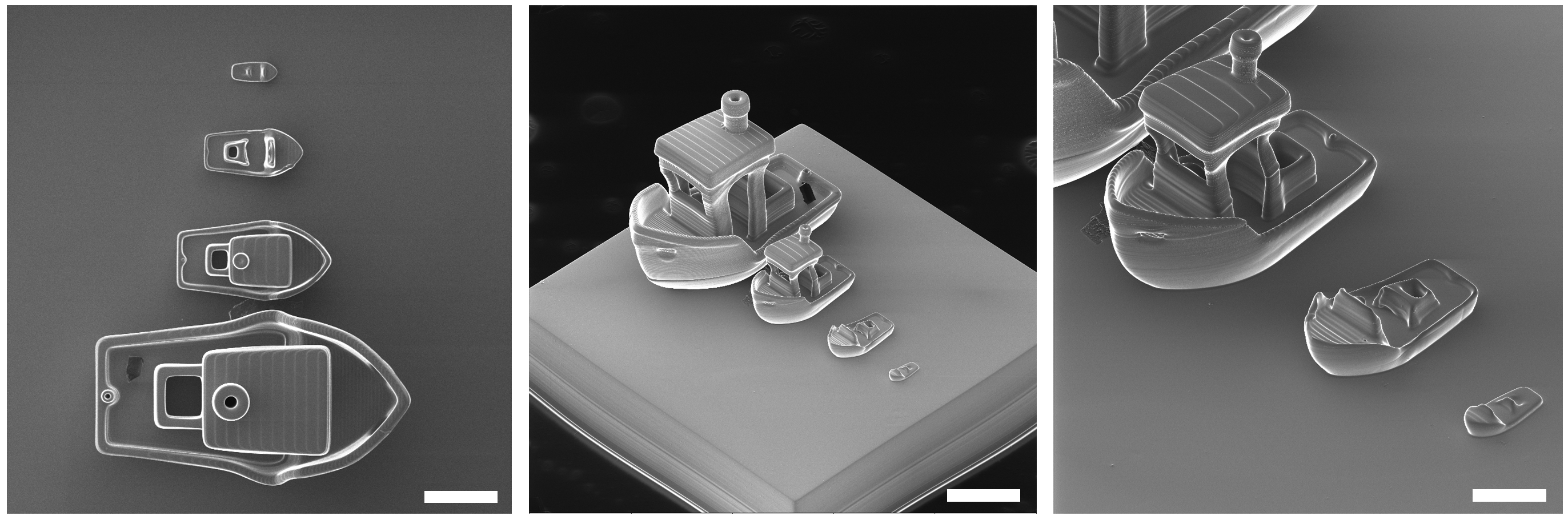


Fig. S5 Array of benchy boats with decreasing size. (A) top view scale bar 100 μm. (B) side view scale bar 100 μm. (C) zoomed view scale bar 40 μm


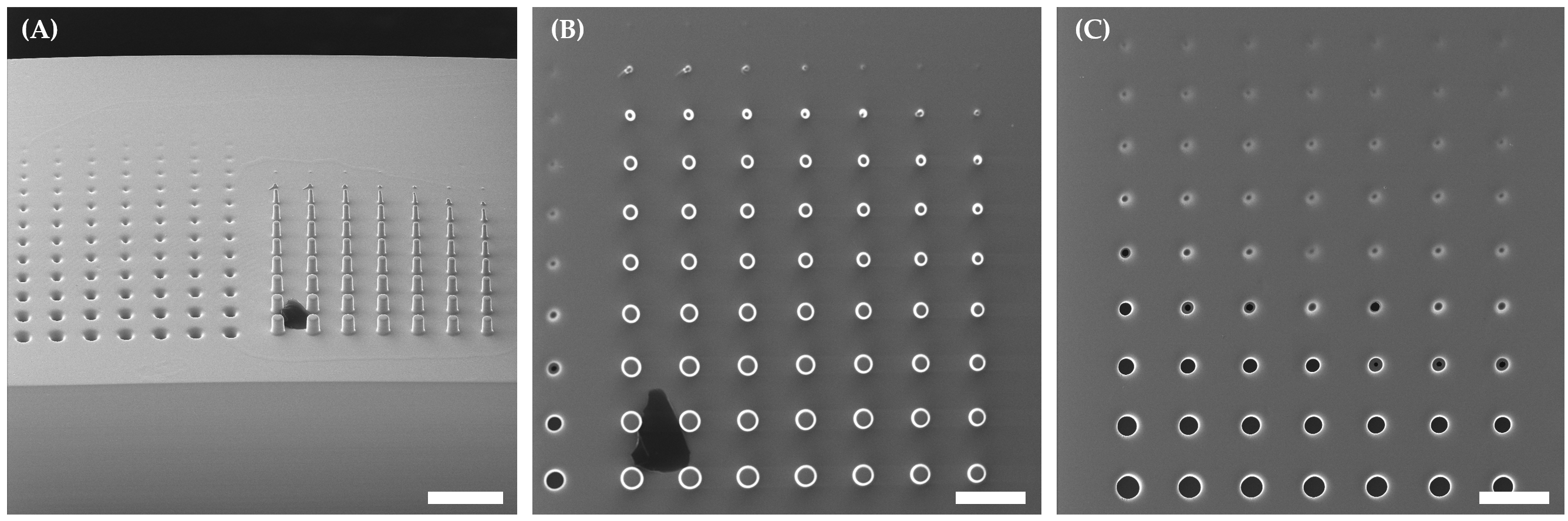


Fig. S6. External and internal feature matrix. (A) scale bar 100 μm. (B-C) scale bar 50 μm.


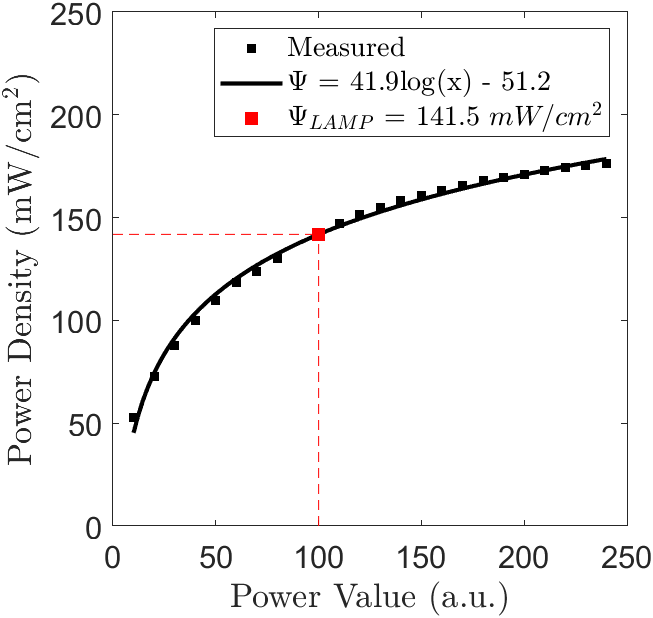


Fig. S7. Optical power as a function of power value setpoint. The LAMP system enables precise control of the optical power output as a function of an arbitrary setpoint between 0 – 240. Measured represents the optical power as determined using power meter (Thorlabs, PM100D). $\boldsymbol{\Psi}$ and $\boldsymbol{\Psi}_{\boldsymbol{LAMP}}$ represent the curve fit and current power measurement used in this work respectively.


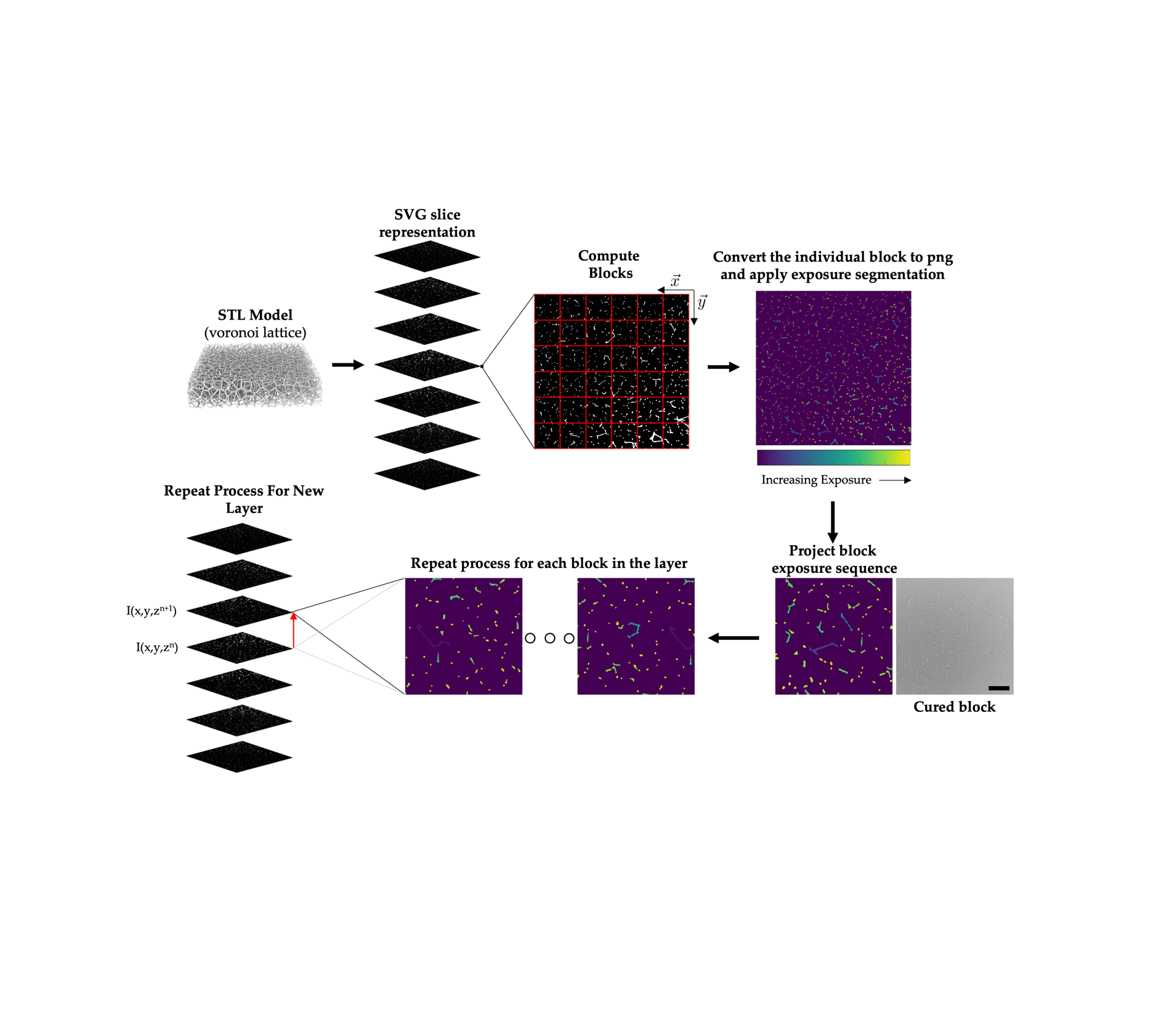


**FIG. S8.** **Process flow example of how the LAMP control software takes a desired STL file (voronoi lattice) and segments it for printing.** (1) the STL file is converted into a SVG representation to enable scaling without data loss. (2) the control software computes the required ‘blocks’ needed to produce each layer and determines the minimum path. (3) the software determines the required modification to the exposure dose depending on the feature size. (4) The required ‘block’ is converted to .png format required for projection, along with its accompanying exposure profile and projected into the resist. A microscope image of the projected block is highlighted in the figure, scale bar 150 μm. (5) this process is repeated for all available blocks in the layer. (6) this procedure is repeated for all required layers in the model file.


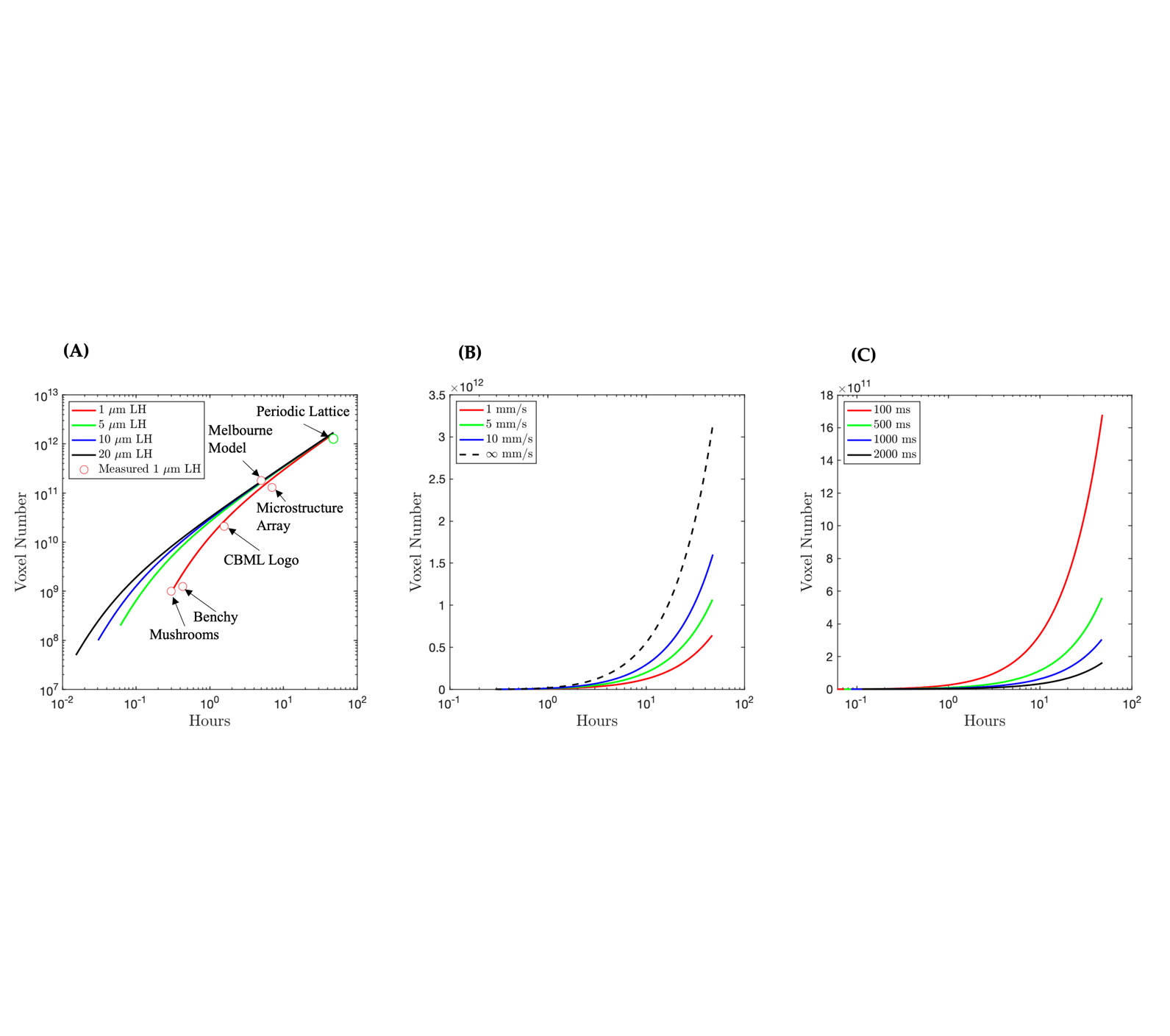


Fig. S9. Effect of print parameters on print time applied to a cubic geometry. (A) effect of varying the layer height on the total print time. Parameters held constant were $\vec{\boldsymbol{v}}$ = 10 mm/s, $\boldsymbol{t}_{\boldsymbol{exposu}\boldsymbol{re}}$ = 400 ms, $\boldsymbol{t}_{\boldsymbol{layer-change}}$ = 4000 ms. Red and green circles indicate locations of actual printed structures. (B) effect of varying the stage velocity on print time, $\boldsymbol{t}_{\boldsymbol{exposure}}$ = 400 ms, $\boldsymbol{t}_{\boldsymbol{layer-change}}$ = 4000 ms, LH = 1μm. (C) effect of varying the exposure time on total print time. $\vec{\boldsymbol{v}}$ = 10 mm/s, $\boldsymbol{t}_{\boldsymbol{layer-change}}$ = 4000 ms, LH = 5μm

**w**

**
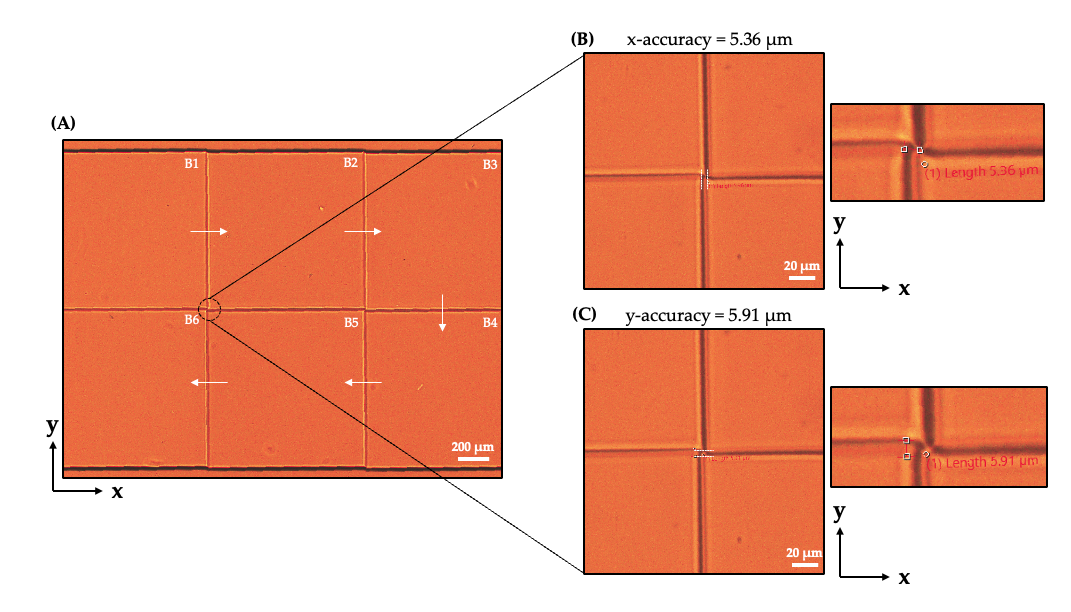
**

Fig. S10. Block alignment and stitch accuracy for the LAMP system. (A) shows a 3000 µm x 2000 µm section projected into a single droplet of resin. (B) Measurement of the stitch accuracy in the x-direction. (C) Measurement of the stitch accuracy in the y-direction.

Movie S1.

Example of a representative cross-section of an object projected into a droplet on a glass coverslip. The object is approximately 10 x 10 mm.
